# The Efficacy and Safety of Transvaginal Ethanol Sclerotherapy in the Treatment of Endometrial Cysts—A Systematic Review

**DOI:** 10.3390/ijms25021337

**Published:** 2024-01-22

**Authors:** Karolina Frankowska, Izabela Dymanowska-Dyjak, Monika Abramiuk, Grzegorz Polak

**Affiliations:** 1 Student Scientific Association, Independent Laboratory of Minimally Invasive Gynecology and Gynecological Endocrinology, Medical University of Lublin, 20-059 Lublin, Poland; k.frankowska10@gmail.com; 2 Independent Laboratory of Minimally Invasive Gynecology and Gynecological Endocrinology, Medical University of Lublin, 20-059 Lublin, Poland; i.dymanowska@gmail.com (I.D.-D.); monika.abramiuk@umlub.pl (M.A.)

**Keywords:** endometriosis, endometrial cyst, ethanol sclerotherapy

## Abstract

Endometriosis, as a chronic disorder that is a source of severe pain ailments and infertility, requires a comprehensive therapeutic approach. Sclerotherapy, consisting of the administration of sclerosing agents into the cyst, is a constantly evolving minimally invasive treatment method for this disease. Hence, the main objective of this systematic review was to evaluate the impact of its most often used variant, transvaginal ethanol sclerotherapy, on endometriosis-related symptoms, endometrial cyst recurrence rate, ovarian reserve, assisted reproductive technology (ART) outcomes, and pregnancy outcomes, as well as to assess potential complications resulting from this treatment. This systematic review was undertaken using PubMed, Scopus, Web of Science, and Cochrane Library databases on 24 November 2023. The risk of bias in included studies was assessed with the use of the Newcastle–Ottawa scale (NOS) and the revised Cochrane risk of bias 2.0 tool for randomized controlled trials. From the 1141 records obtained from all databases, 16 studies have been included in this review. The use of ethanol sclerotherapy was characterized by a low rate of post-procedural complications. The recurrence rate of endometrial cysts after the procedure depended on the ethanol instillation time within the cyst. Although ethanol sclerotherapy had negligible influence on ovarian reserves when compared to laparoscopic cystectomy, the effects of both these methods on pregnancy outcomes were comparable. This review identifies that sclerotherapy is safe, provides significant relief of symptoms, and does not impair the reproductive potential of the patients.

## 1. Introduction

Endometriosis, defined as a benign estrogen-dependent disease that affects mainly women of childbearing age, typically manifests in a triad of symptoms including dysmenorrhea, dyspareunia, and impaired fertility [1,2,3]. Histologically, it consists of the pathological placement of the endometrial tissue in the extra-uterine locations. Although almost all organs may harbor ectopic lesions, the implants are most often found within the pelvic cavity compartment. There, endometrial tissue forms both lesions on the peritoneal surface, as well as those located within the deep structures of the pelvis and in the ovaries [4,5]. In the latter case, endometrial cysts found within the surface of the ovary structure are filled with endometrial debris and blood [6]. Such cyst content creates a highly prooxidant environment, which negatively affects ovarian follicles located in the vicinity of the pathological tissue [7]. Therefore, the presence of endometrial cysts may per se harm the ovarian reserve [8,9]. Treatment of ovarian endometriosis varies depending on its principal expected effects, which are pain alleviation or fertility restoration; however, for both indications, pharmacotherapy and surgical management are included [10]. Over the years, researchers have raised their concern about unintentional damage of normal ovarian tissue in the course of laparoscopic cyst excision, resulting in the subsequent deterioration of ovarian reserve [11,12,13]. Thus, the current literature emphasizes the need for novel endometriosis management which should consider the chronic nature of the disease and the abandonment of repeated surgeries [14].

In accordance with the idea of such a modified perception of the endometriosis treatment, the growing prevalence of management involving the aspiration of the cyst content and the following injection of some sclerosing agents has been observed. In general, its mechanism of action is aimed at the sclerotic closure of the cyst cavity and its following occlusion [15]. First, sclerotherapy in the treatment of ovarian endometriosis was introduced over 30 years ago [16]. Since then, several substances, such as ethanol [17], methotrexate [18], interleukin-2 (IL-2) [19], or tetracycline [20], as well as various technical variants of the method [21,22,23], have been proposed to use; however, transvaginal sclerotherapy involving ethyl alcohol remains the most commonly applied variation of this technique [24]. 

Therefore, there is an ongoing need for evaluation of this treatment procedure. Hence, we decided to conduct an up-to-date systematic review aiming to assess the safety and efficacy of transvaginal ultrasound-guided ethanol sclerotherapy in the treatment of ovarian endometrioma. In the course of our study, we have focused on the influence of this treatment method on endometriosis symptoms, ovarian reserve, assisted reproductive technology (ART) outcomes, pregnancy outcomes, the recurrence rate of endometrial cysts, and complications resulting from this method. Additionally, some individual subparagraphs have compared the outcomes of sclerotherapy with laparoscopic cystectomy.

## 2. Methods

### 2.1. Study Design

The reporting of this systematic review was conducted in accordance with the standards of preferred reporting items for systematic review and meta-analysis (PRISMA) Statement [25].

### 2.2. Eligibility Criteria

#### 2.2.1. Inclusion Criteria

Inclusion criteria for this systematic review were as follows: (1) studies evaluating the use of transvaginal ultrasound-guided ethanol sclerotherapy in patients diagnosed with ovarian endometrioma, (2) manuscripts peer-reviewed, and (3) manuscripts written in English.

#### 2.2.2. Exclusion Criteria

Exclusion criteria for this systematic review were as follows: (1) manuscripts that did not investigate the role of transvaginal ultrasound-guided ethanol sclerotherapy in ovarian endometrioma, (2) manuscripts that evaluated only ultrasound features of the lesions, or other parameters not linked to the aim of this study, (3) document types including review, systematic review, meta-analysis, technical report, letter, editorial, and conference summary, and (4) manuscripts with non-available full-text.

### 2.3. Literature Search Strategy

A systematic search was performed on 24 November 2023 with the use of PubMed, Web of Science, Scopus, and Cochrane Library databases. Two researchers (K.F. and I.D.-D.) conducted the database review independently. The discrepancies between the authors were regularly consulted and resolved in consultation with G.P. No time restrictions for searching articles in databases were applied. The following search string was used: “ovarian endometrioma” OR “OMA” OR “endometrial cyst” OR “ovarian endometriosis” OR “chocolate cyst” AND “ethanol sclerotherapy” OR “transvaginal ethanol sclerotherapy” OR “ultrasound-guided ethanol sclerotherapy” OR “US-guided ethanol sclerotherapy” OR “transvaginal ethanol injection” OR “ultrasound-guided ethanol injection” OR “US-guided ethanol injection”.

### 2.4. Selection Process and Data Extraction

The article selection process was conducted in three stages. First, the records were screened and all non-English written papers and book chapters were excluded. Secondly, the titles and abstracts of the articles were independently assessed by two reviewers to evaluate whether they met the inclusion criteria. Furthermore, the selected publications were entirely read and a definitive decision on the article eligibility was made. No automation tools were used during all stages of the process. Data contained in the included records were manually extracted from the main text. No automation tools have been used in this process.

### 2.5. Quality Assessment and Data Synthesis

Two independent researchers (I.D.-D. and M.A.) assessed the quality of eligible studies. The Newcastle–Ottawa scale (NOS) was applied to case-control and cohort studies [26]. Each article could receive a maximum of 9 points on this scale; 3, 2, and 4 points were maximally awarded in the following categories: selection, comparability, and outcomes, respectively. The quality of randomized studies was evaluated with the use of the revised Cochrane risk of bias 2.0 tool [27]. This tool evaluates studies in the following five domains: randomization process, deviations from the intended interventions, missing outcome data, measurement of the outcome, and selection of the reported result. Each domain was given a “+” or “−”. All discrepancies between the authors were discussed with the G.P. and resolved through a common consensus. The data obtained from each eligible study were synthesized within the main text of the paper and corresponding Tables.

## 3. Results

### 3.1. Study Selection

**Figure 1:**
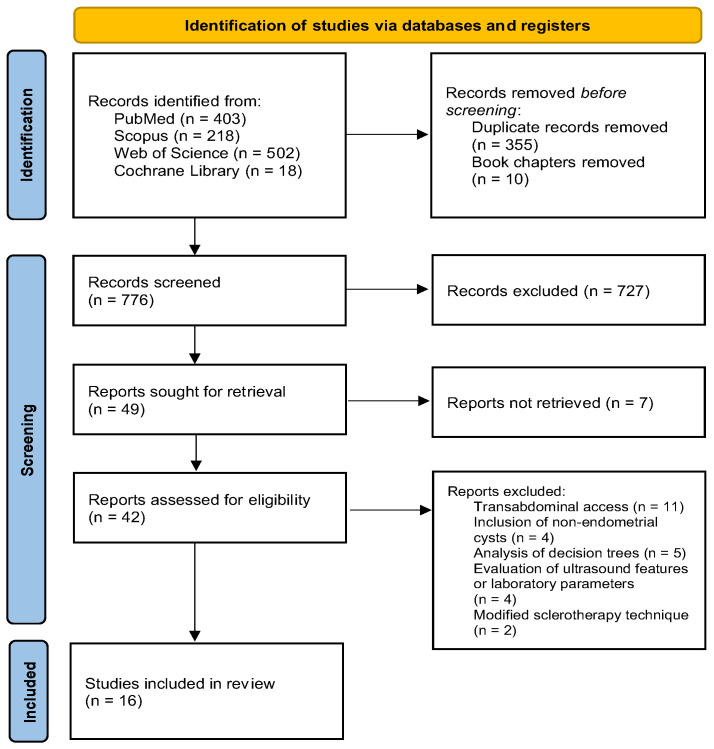
A flowchart presenting the process of study selection in accordance with PRISMA guidelines.

The total number of articles searched in all databases was 1141. After removing the duplicates, 786 publications were further evaluated with regard to suitability for analysis. In a further step, 10 records were removed before the screening as they were book chapters and additionally, 80 records were eliminated due to the language of the paper other than English. Therefore, 696 records were identified as relevant for further screening. After reading the titles and abstracts of all records, 647 were recognized as failing to meet eligibility criteria and were excluded from further analysis. In addition, 7 records could not be retrieved. Therefore, the final step of the literature screening included a full-text reading of 42 articles that previously were included as eligible for further analysis. The final assessment of papers resulted in the inclusion of 16 articles [28,29,30,31,32,33,34,35,36,37,38,39,40,41,42,43]. The detailed reasons for the exclusion of other articles after full-text reading and all details regarding the prior study selection process are presented on the Prisma flowchart (Figure 1).

### 3.2. Results of Individual Studies

The overall characteristics of the evaluated studies are presented in Table 1.

#### 3.2.1. Impact on Disease Symptoms

Endometriosis is a disease presenting a heterogeneous nature of associated symptoms; however, it is observed that most patients suffer from dysmenorrhea, chronic pelvic pain, and dyspareunia. In addition, the disease may be less frequently accompanied by pain or discomfort in other locations [3]. Although endometriosis rarely remains asymptomatic [44], only 5 of the 16 included studies evaluating the changes in the severity of symptoms that have occurred after sclerotherapy [32,34,36,40,42]. To measure changes in pain intensity, two authors adopted numerical scales, where pain intensity was scored from 1 to 10 points. Aliabad et al. noticed a significant reduction in dysmenorrhea (*p* < 0.001) and dyspareunia (*p* < 0.001) [32]. Similarly, Hsieh et al., by using the same research tool, found significant alleviation in dysmenorrhea, dyspareunia, and chronic pelvic pain after the sclerotherapy procedure (*p* < 0.05) [34]. In addition, the same authors did not notice any differences in pain alleviation between the groups in which the alcohol was aspirated or left within the cyst [34]. In the other studies, a reduction in symptoms was demonstrated without the use of any research tools. In a study by Ikuta, 15 of 18 patients (83.33% of patients) reported lower dysmenorrhea and pelvic pain in the post-sclerotherapy period [36]. Noma et al. noticed the reduction in dysmenorrhea in 13 of 20 patients (65% of patients), a reduction in pelvic pain in 10 of 14 patients (71.43% of patients), and a reduction in backache in 1 affected patient (full effectiveness) [40]. The least satisfactory results regarding changes in symptoms were obtained by Vaduva et al. [42], as the authors observed relief of pain and discomfort in less than half of patients who underwent sclerotherapy (26 of 54 patients—48%). 

#### 3.2.2. Impact on Ovarian Reserve

Ovarian reserve, which reflects the reproductive potential of the ovaries, could be expressed as an antral follicle count (AFC) or as various hormonal measurements including antimüllerian hormone (AMH), follicle-stimulating hormone (FSH), estradiol, and inhibin B values [45]. Ten of the included studies assessed the impact of sclerotherapy on ovarian reserve [28,30,32,33,34,35,37,38,41,42]. In the analyzed studies, the concentrations of AMH and AFC were the most often applied parameters. Almost none of the studies showed significant changes in AMH levels after sclerotherapy [30,32,33,41,42]. The only report that presented inconsistent results was that conducted by Huang et al., as the authors noticed a significant AMH decrease in a group with retained ethanol; however, no changes were observed when the sclerosing agent was aspirated after 3 min of retention [35]. Although in the above-mentioned studies the post-procedural AMH measurements were performed at three months [32,33,42], six months [35], twelve months [41], or even seven years [30] after sclerotherapy, the tendency of unaltered AMH was maintained. The additional juxtaposing of the AMH values after the sclerotherapy procedure with measurements of AMH after laparoscopic cystectomy revealed a substantial decline of AMH in the case of the surgery [41,42]. 

All of the studies evaluating the impact of sclerotherapy on AFC found favorable effects of sclerotherapy on this marker of ovarian reserve [32,34,38]. In both three- [32] as well as twelve-month [34] intervals, a substantial increase in AFC was observed. Contrary to the above-mentioned results for AMH, the AFC values did not differ depending on the usage of the alcohol aspiration or retention [34]. Moreover, comparing AFC in patients who underwent sclerotherapy and laparoscopic cystectomy revealed its greater values within the first-mentioned group [38]. 

The other rarely-evaluated marker, which was FSH, also did not change significantly one [37] or three months [28,33] after the sclerotherapy.

#### 3.2.3. Impact on Assisted Reproductive Technology (ART) Outcomes

Although numerous studies have focused on the impact of sclerotherapy on ART outcomes, the multitude of parameters used as indicators of the procedure’s success renders them difficult to juxtapose. In total, 7 of the 16 papers described the impact of sclerotherapy on ART outcomes [28,30,31,37,38,39,43] and the results of these researches have been summarized in Table 2 and Table 3. 

In these studies, the non-uniformity of applied ovarian stimulation protocols during ART could be observed (Table 2). The comparison of parameters regarding oocyte number and their quality predominantly included the mean values of the total number of oocytes retrieved and the number of mature oocytes, which ranged from 3.95 to 12.4 [28,30,31,37,38,43] and from 5.5 to 10.5 [28,30,38,39,43], respectively (Table 2). The results by Lee et al. and Yazbeck et al. have also revealed significantly higher values of these parameters after sclerotherapy treatment when compared to laparoscopic cystectomy [38,43]; however, in other studies, the assessment of oocytes received for ART procedures between women who underwent sclerotherapy and laparoscopic cystectomy or women who did not undergo any interventions have not brought any difference [28,30,37,39].

Nevertheless, the above-described correlations were not reflected in the parameters measuring the number and quality of obtained embryos, as well as their transfer (Table 3). 

The number of total embryos, the cryopreserved embryos, diploid embryos, and “top embryos” obtained during ART was comparable between patients who underwent sclerotherapy and those non-treated [28,39], similarly to the number of total embryos between patients who underwent sclerotherapy compared to the laparoscopy groups [30]. There was also no difference between such groups in parameters describing embryo transfer [28,38,39,43].

The patients who underwent sclerotherapy displayed rather similar values of fertilization and implantation rates, which ranged from 60.8% to 63.06% [28,37,39,43] and from 21 to 31.5% [39,43], respectively. Also, both these rates shared comparable ranges when the group that underwent sclerotherapy was juxtaposed with patients who did not undergo any procedure [28,37,39] or with the group that underwent laparoscopic cystectomy [43]. The values of fertilization and implantation rates, as well as the intergroup differences, are presented in Table 3.

#### 3.2.4. Impact on Pregnancy Outcomes

While some of the included studies described the outcomes of applied ART, others reported the number of pregnancies from natural conception and others reported these data cumulatively. In total, 13 of the 16 included studies reported pregnancy outcomes after sclerotherapy [28,29,30,31,33,34,35,36,37,38,39,40,43]. The percentage of spontaneous pregnancies after sclerotherapy was divergent as it ranges from 8.16% to 52.1% [34,36,40]. In addition, it has been proven that undergoing ethanol sclerotherapy increases the chance of spontaneous pregnancy in comparison to laparoscopic cystectomy [40]. 

Comparisons of pregnancies obtained by ART after an ethanol sclerotherapy procedure were made by using various parameters such as the total number of pregnancies, chemical pregnancy rate, ongoing pregnancy rate, cumulative pregnancy rate, clinical pregnancy rate, or live birth rate [28,30,31,33,37,38,39,43]. In general, the range of values in which the results were contained was diverse, even if each of the parameters defining pregnancy was analyzed separately (Table 4). Of the studies comparing the number of pregnancies achieved with ART between groups of patients who underwent sclerotherapy and laparoscopy or patients who were not subjected to any procedures [28,30,37,38,39,43], only Miquel et al. [39] and Yazbeck et al. [43] have shown a statistically significant advantage of sclerotherapy over laparoscopy or non-intervention, respectively (Table 4).

The current literature was conflicting in evaluating the effect of alcohol instillation time on the number of achieved pregnancies. Both Huang et al. and Aflatoonian et al. cumulatively compared the number of spontaneous pregnancies and pregnancies being the result of ART between groups in which there was alcohol aspiration and ethanol retention [29,35]. Huang et al. found that ethanol aspiration favored a greater pregnancy rate [35], while Aflatoonian et al. found no differences between both groups in terms of chemical pregnancy rate, ongoing pregnancy rate, and live birth rate [29].

#### 3.2.5. Recurrence of Ovarian Cysts

In general, 14 studies assessed the recurrence of endometrial cysts after sclerotherapy treatment [28,29,30,31,33,34,35,36,37,39,40,41,42,43]. Nevertheless, the evaluation of this parameter was difficult to conduct because the authors adopted different criteria for recurrence. Thus, different times to recurrence, ranging from 3 months to 7 years, and different minimum sizes of lesions considered as a recurrence, which amounted from 2 through 3 cm, were adopted. Moreover, some of the studies did not provide any detailed criteria for recurrence. In general, the recurrence rate ranged from 0% to 62.5% [28,29,30,31,33,34,35,36,37,39,40,41,42,43]. Several studies have compared the recurrence rate between ethanol aspiration and ethanol retention groups [29,34,35] and the recurrence rate values depending on different ethanol instillation times [40]. Hsieh et al. and Huang et al. have shown that retention of ethanol inside the cyst interior reduced the risk of recurrence [34,35]. Similarly, Noma et al. observed that even prolonging the ethanol instillation time above or equal to 10 min with its subsequent aspiration could significantly reduce recurrences compared to a shorter instillation period [40]. 

Among the studies comparing the recurrence of cysts after sclerotherapy and after laparoscopic cystectomy [30,40,41], only one conducted by Alborzi et al. has found a significant difference in recurrence rate between these two groups and noticed a higher recurrence rate in patients who underwent sclerotherapy [30]. 

The phenomenon of recurrence depends also on post-operative pharmacotherapy [10]. Nevertheless, in only four analyzed studies, the patients received post-sclerotherapy pharmacological treatment [33,34,36,40]. While in all studies some patients received treatment with gonadotropin-releasing hormone (GnRH) agonists [33,34,36,40], some authors also proposed the use of oral contraceptives [33,34] or danazol [34]. In addition, the indications for post-operative pharmacotherapy and the time of its duration have differed [33,34,36,40]. Thus, the effect of such treatment on the cyst recurrence rate remains difficult to assess.

#### 3.2.6. Occurrence of Post-Procedural Complications

Fourteen of the included studies referred to the occurrence of post-sclerotherapy complications [28,29,30,31,32,33,34,35,36,37,39,40,42,43]. In addition, most authors apply the division into major and minor ones to describe their results more precisely. Eight studies did not report any minor or major complications, hence suggesting the high safety of the sclerotherapy treatment [28,29,30,31,32,33,35,37]. Other authors typically reported few or even single complications in groups of women after sclerotherapy; however, the percentage of patients affected by complications did not exceed 12 percent in each study. The complications mainly manifested as a fever [36], abdominal and pelvic pain [42,43], or co-occurrence of these symptoms [39], as well as the occurrence of abscesses [39] or pelvic inflammation [34].

The only study in which the percentage of post-sclerotherapy complications have exceeded 25% of patients was conducted by Noma et al. Although no major complications have been observed, the percentage of minor ones was high and reached 27% of patients (20 of 74 patients). The main reported was abnormal bleeding; however, the patients also experienced lower abdominal pain and alcohol intoxication [40]. 

#### 3.2.7. Quality and Publication Bias Assessment

For the assessment of the quality of 14 of the included studies, we used NOS. Table 5 presents the assessment of the quality of the included studies with the application of this tool. The scores received by the articles ranged from 6 to 8 points. Most studies reached the seven-point threshold, classifying them as studies with a low risk of bias. In addition, the risk of bias in two studies, conducted by Aflatoonian et al. [28] and by Tehrani et al. [41], was evaluated with the revised Cochrane risk of bias 2.0 tool due to their randomized trials design. The studies by Aflatoonian et al. [28] and Tehrani et al. [41] received a positive rating in all five assessed subcategories, which corresponds with a low risk of bias. 

## 4. Discussion

In recent decades, the diagnosis and treatment regimens for endometriosis have constantly evolved. The beginning of understanding endometriosis as a systemic and chronic disease has entailed several substantial changes [46,47]. The key ones included the departure from laparoscopy as the gold standard in the diagnosis of the disease and calling for a more balanced approach to the use of surgical treatment [10,14]. Therefore, the use of minimally invasive methods of endometriosis treatment, such as sclerotherapy, is a relevant answer to these new paradigms.

An important advantage of sclerotherapy, presented in our review, is the low invasiveness of this method. The absence of serious side effects and the sporadic appearance of minor post-procedural complications make this procedure a safe alternative to laparoscopic cystectomy, which is in turn characterized by a higher risk of more severe complications [48]. 

Another satisfactory effect of ethanol sclerotherapy concerns the low recurrence rate of endometrial cysts. Moreover, an observed tendency for a lower recurrence rate when longer instillation times or ethanol retention was applied may be valuable for clinicians to use in daily practice. This relationship may be explained by increased cyst fibrosis under the influence of longer alcohol instillation time [49]. Nevertheless, the crucial future objective should be to be precise about the possible side effects resulting from ethanol retention.

Our analysis has also shown the satisfactory efficacy of sclerotherapy in relieving the main symptoms of endometriosis. Since pain is one of the flagship symptoms of ovarian endometrial cysts [50], our significant observation is the effectiveness of sclerotherapy in its reduction. This proves that this technique may be useful for patients primarily suffering from pain symptoms. 

On the other hand, it is crucial to determine the benefits of sclerotherapy for infertile patients, especially taking into consideration that laparoscopic cystectomy may reduce the values of reproductive potential markers [12,13]. According to the observations from our review, ethanol sclerotherapy has not reduced the values of markers of ovarian reserve such as AMH, AFC, or FSH. In addition, we noticed improved ovarian reserve in patients who underwent sclerotherapy in comparison to those who underwent laparoscopic cystectomy. Based on these observations, it can be concluded that sclerotherapy exerts no detrimental effects on ovarian function. Furthermore, this conclusion seems to be supported by a mostly beneficial effect of sclerotherapy on the number of oocytes retrieved compared to the effect exerted by laparoscopic cystectomy. This is a favorable outcome of sclerotherapy, particularly in light of reports indicating endometriosis as a factor able to impair the process of oocyte retrieval [51]. Additionally, in the course of the research, we observed rather a lack of differences in pregnancy outcomes between groups of patients who underwent sclerotherapy and those who underwent laparoscopic cystectomy or who were untreated. Therefore, we hypothesized that some alterations within the eutopic endometrium of women with endometriosis may be the reason for its impaired condition and poorer pregnancy outcomes [52,53]. 

Although, in general, transvaginal sclerotherapy could be considered a useful method, its limitations should be also acknowledged. This procedure does not allow for visualization of the entire abdominal cavity and treatment of endometriotic lesions outside of the ovaries. This is an important limitation as isolated endometrial cysts without concomitant endometriotic lesions in other localizations are a rarity [54].

As we mentioned above, the use of sclerotherapy in endometriosis treatment is not restricted to one technique and it includes several variations starting from laparoscopic access to the use of the transvaginal route through the application of different sclerosing agents, their different concentrations, and different instillation times [55]. The recently published instructional video article aimed at popularizing the method demonstrated the transvaginal sclerotherapy with 10-min instillation of high-percentage ethanol [21]. Taking into consideration that, in most studies, this variation of sclerotherapy has already been investigated, we have limited our evaluation to this method.

Our review aimed to select studies with as close as possible methodological background. Therefore, the studies in which some sclerotherapy procedures were conducted by transabdominal access or in which ethanol was combined with other sclerosing agents have been initially excluded. Nevertheless, we could not avoid all discrepancies regarding the methodology of the included studies and our systematic review is not free of some limitations. First, the authors used different ethanol concentrations ranging from 20% to 98%. So far, no studies have compared the results or complications of endometrial cyst ethanol sclerotherapy at different ethanol concentrations. The current literature suggests that higher ethanol concentrations were more efficient in their sclerosing action, however, these studies were conducted in vitro [56] and on a small group of patients having hepatic cysts [57]. Thus, this issue certainly needs to be explored in the future on the endometrial cyst model for further standardization of the sclerotherapy procedure. Similarly, the evaluation of the most efficient time of ethanol instillation within the cyst should be further conducted in association with the selection of the most suitable ethanol concentrations. 

Secondly, the application of different ovarian stimulation protocols in studies assessing the impact of sclerotherapy on the results of ART could affect the obtained results [58]. Thus, this should be taken into account in future studies.

Moreover, the post-sclerotherapy pharmacological therapy, which was applied in some patients, may also affect the evaluation of pain or recurrence rate [59]. According to European Society of Human Reproduction and Embryology (ESHRE) guidelines, pharmacotherapy, as a post-surgical treatment option, aims to prevent the suppression of the lesions and to provide pain alleviation [10]. Therefore, such therapeutic management may alter the recurrence rate and severity of symptoms reported after the sclerotherapy procedure. Although Noma et al. found that the implementation of hormonal treatment after sclerotherapy did not affect the recurrence rate in their study, [40] this topic should still be under assessment.

## 5. Conclusions

Sclerotherapy is a safe minimally invasive method of treating endometrial cysts. The prolonged instillation time of ethanol inside the cyst or its retention seems to influence the risk of cysts’ recurrence. Sclerotherapy could be considered an effective procedure for managing endometriosis-associated symptoms. On the one hand, this treatment method has reduced pain symptoms. On the other, it is also suitable for infertile women, as the negligible effect of the procedure on the ovarian reserve has been observed. Although the impact of endometrial cyst sclerotherapy on pregnancy outcomes most often was not improved in comparison to the effects of laparoscopic cystectomy or lack of interventions, sclerotherapy could be also considered as a promising treatment method not impairing reproductive outcomes.

## Figures and Tables

**Table 1:** Tabular representation of general characteristics of the included studies.

Ref.	Study	Year	Country	Study Design	Study Groups Characteristics	Details of the Procedure
[28]	Aflatoonian et al.	2013	Iran	prospective randomized clinical trial	40 patients with recurrent ovarian endometriomas who underwent IVF including 20 patients who underwent sclerotherapy and 20 patients without any previous interventions	transvaginal access; use of 98% ethanol; filling 80% of the initial cyst volume; total aspiration after 10 min
[29]	Aflatoonian et al.	2020	Iran	retrospective study	38 women with recurrent ovarian endometriomas including 25 women who underwent sclerotherapy with ethanol aspiration after 10 min and 13 patients who underwent sclerotherapy with ethanol retention	transvaginal access; use of 95% ethanol; filling 2/3 of the initial cyst volume; aspiration after 10 min or alcohol retention
[30]	Alborzi et al.	2021	Iran	prospective cross-sectional study	101 infertile patients who underwent ART including 44 patients who underwent sclerotherapy at the time of oocyte retrieval and 57 patients who underwent LPS one year before ART	transvaginal access; use of 96% ethanol; filling 80% of the initial cyst volume; ethanol retention
[31]	André et al.	2011	Brazil	prospective pilot study	21 patients with recurrent ovarian endometrial cysts who underwent sclerotherapy and the following controlled ovarian hyperstimulation (COS)	transvaginal access; use of ethanol; filling 70% of the initial cyst volume; total aspiration after 5 min
[32]	Anvari et al.	2023	Iran	prospective clinical trial	48 patients including 23 patients who underwent ethanol sclerotherapy and 25 healthy controls	transvaginal access; use of 98% ethanol; filling 2/3 of the initial cyst volume; ethanol retention
[33]	Begum et al.	2015	Bangladesh	prospective study	53 infertile patients with recurrent ovarian endometriomas who underwent ethanol sclerotherapy	transvaginal access; use of 95% ethanol; filling 75% of the initial cyst volume; ethanol retention and substantial retention of 5–10 mL of ethanol
[34]	Hsieh et al.	2009	Taiwan	retrospective study	108 patients with recurrent ovarian endometriomas including 78 patients who underwent sclerotherapy with ethanol aspiration and 30 patients who underwent sclerotherapy with ethanol retention	transvaginal access; use of 95% ethanol; filling 80% of the initial cyst volume; total ethanol aspiration after maximum 10 min or alcohol retention
[35]	Huang et al.	2021	Taiwan	retrospective study	124 patients including 44 patients who underwent sclerotherapy with a retention of 3–10 mL of ethanol in situ and 80 patients who underwent sclerotherapy with ethanol aspiration after 1–3 min	transvaginal access; use of 95% ethanol; filling volume depended on the initial cyst size; aspiration after 1–3 min or retention of 3–10 mL of alcohol
[36]	Ikuta et al.	2006	Japan	retrospective study	18 patients who underwent ethanol sclerotherapy	transvaginal access; use of absolute ethanol; total aspiration after 5 min
[37]	Koike et al.	2002	Japan	retrospective study	110 patients including 45 subfertile patients with ovarian endometrioma who underwent sclerotherapy and 65 subfertile patients without ovarian endometrioma	transvaginal access; use of 50% ethanol; aspiration after 5 min
[38]	Lee et al.	2014	Korea	retrospective study	101 patients who underwent IVF including 29 patients who underwent sclerotherapy, 36 patients who underwent LPS, and 36 patients without any previous interventions	transvaginal access; use of 20% ethanol; filling 80–90% of the initial cyst volume; total aspiration
[39]	Miquel et al.	2020	France	single-center retrospective cohort study	74 patients who underwent IVF with ultra-long-agonist protocol including 37 patients who underwent ethanol sclerotherapy and 37 patients with ovarian endometrioma without any previous interventions	transvaginal access; use of 96% ethanol; filling 60% of the initial cyst volume; total aspiration after 10 min
[40]	Noma et al.	2001	Japan	retrospective study	100 patients including 74 patients who underwent ethanol sclerotherapy and 26 patients who underwent LPS	transvaginal access; use of pure ethanol; filling 80% of the initial cyst volume; total aspiration after mean 8.6 min in recurrent endometriomas and 14.5 min in non-recurrent endometriomas
[41]	Tehrani et al.	2022	Iran	randomized double-blind clinical trial	70 patients including 35 patients who underwent sclerotherapy and 35 patients who underwent LPS	transvaginal access; use of 95% ethanol; filling 80% of the initial cyst volume; total aspiration after 20 min
[42]	Vaduva et al.	2023	Romania	retrospective study	96 patients including 54 patients who underwent sclerotherapy and 42 patients who underwent LPS	transvaginal access; use of 96% ethanol; filling 60% of the initial cyst volume; total aspiration after 7 min
[43]	Yazbeck et al.	2009	France	prospective comparative study	57 patients with recurrent endometriotic cysts who underwent ART, including 31 patients who underwent sclerotherapy and 26 patients who underwent LPS	transvaginal access; use of pure ethanol; filling 80% of the initial cyst volume; total aspiration after 10 min

Abbreviations: LPS—laparoscopic cystectomy; IVF—in vitro fertilization.

**Table 2:** Characteristics of the studies regarding ART outcomes (oocyte characteristics) amongst patients with endometrial cysts treated with ethanol sclerotherapy.

Ref.	Study	Ovarian Stimulation Protocols	Total Number of Retrieved Oocytes	Number of Mature Oocytes
[28]	Aflatoonian et al.	long protocol with GnRH agonist	in the sclerotherapy group: 7.83; in the control group: 7.55; *p* = NS	in the sclerotherapy group: 6.11; in the control group: 5.45; *p* = NS
[30]	Alborzi et al.	protocol with GnRH antagonist	in the sclerotherapy group: 7.95 in the LPS group: 6.11; *p* = NS	in the sclerotherapy group: 6.66; in the LPS group: 5.77; *p* = NS
[31]	André et al.	long protocol with GnRH agonist	in the sclerotherapy group: 3.95 per cycle	NR
[37]	Koike et al.	protocol with GnRH analogue	in the sclerotherapy group—8.9; in the control group: 12.4; *p* = NS	NR
[38]	Lee et al.	long protocol with GnRH agonist or protocol with GnRH antagonist	in the sclerotherapy group: 12.4; in the LPS group: 8.2; in the control group: 12.4; *p* = 0.016	in the sclerotherapy group: 10.5; in the LPS group: 6.9; in the control group: 10.7; *p* = 0.010
[39]	Miquel et al.	ultra-long protocol with GnRH agonist	NR	in the sclerotherapy group: 5.5; in the control group: 5.8; *p* = NS
[43]	Yazbeck et al.	ultra-long, long, short, or not specified protocols	in the sclerotherapy group: 11.4; in the LPS group: 7.0; *p* = 0.03	in the sclerotherapy group: 10.4; in the LPS group: 6.1; *p* = 0.02

Abbreviations: GnRH—gonadotropin-releasing hormone; LPS—laparoscopic cystectomy; NR—not reported; NS—not significant.

**Table 3:** Characteristics of the studies regarding ART outcomes (embryo and embryo transfer characteristics, implantation, and fertilization rates) amongst patients with endometrial cysts treated with ethanol sclerotherapy.

Ref.	Study	Number of Total Embryos	Number of Diploid Embryos	Number of Top Embryos	Number of Cryopreserved Embryos	Number of Transferred Embryos	Fertilization Rate	Implantation Rate
[28]	Aflatoonian et al.	in the sclerotherapy group: 4.72; in the control group: 3.8; *p* = NS	NR	NR	NR	in the sclerotherapy group: 2.17; in the control group: 2.35; *p* = NS	in the sclerotherapy group: 63.06%; in the control group: 60.38; *p* = NS	NR
[30]	Alborzi et al.	in the sclerotherapy group: 5.18; in the LPS group: 4.48; *p* = NS	NR	NR	NR	NR	NR	NR
[37]	Koike et al.	NR	NR	NR	NR	NR	in the sclerotherapy group—92/148 (62%); in the control group—279/443 (63%); *p* = NS	NR
[38]	Lee et al.	NR	NR	NR	NR	in the sclerotherapy group: 3.2; in the LPS group: 2.7; in the control group: 2.9; *p* = NS	NR	NR
[39]	Miquel et al.	NR	in the sclerotherapy group—3.4; in the control group—3.4; *p* = NS	in the sclerotherapy group—0.3; in the control group—0.3; *p* = NS	in the sclerotherapy group—0.7; in the control group—0.4; *p* = NS	in the sclerotherapy group—1.9; in the control group—1.7; *p* = NS	in the sclerotherapy group—62.3%; in the control group—58.2%; *p* = NS	in the sclerotherapy group—21%; in the control group—10.7%; *p* = NS
[43]	Yazbeck et al.	NR	NR	NR	NR	in the sclerotherapy group: 2.1 in the LPS group: 1.8; *p* = NS	in the sclerotherapy group: 60.8% in the LPS group: 80.1%; *p* = NS	in the sclerotherapy group: 31.5% in the LPS group: 32.3%; *p* = NS

Abbreviations: LPS—laparoscopic cystectomy; NR—not reported; NS—not significant.

**Table 4:** Characteristics of the studies regarding pregnancy outcomes.

Ref.	Study	Pregnancy Rate	Chemical Pregnancy Rate	Ongoing Pregnancy Rate	Cumulative Pregnancy Rate	Clinical Pregnancy Rate	Live Birth Rate
[28]	Aflatoonian et al.	NR	in the sclerotherapy group: 33.3%, in the control group: 20%; *p* = NS	NR	NR	in the sclerotherapy group: 27.8%, in the control group: 15%; *p* = NS	NR
[30]	Alborzi et al.	NR	NR	NR	NR	in the sclerotherapy group: 34.1%, in the LPS group:42.1%; *p* = NS	in the sclerotherapy group: 29.5%, in the LPS group: 38.6%; *p* = NS
[31]	Andre et al.	in the sclerotherapy group: 20%	NR	NR	NR	NR	NR
[33]	Begum et al.	in the sclerotherapy group: 31.71%	NR	NR	NR	NR	NR
[37]	Koike et al.	in the sclerotherapy group: 30%, in the control group: 40%; *p* = NS	NR	NR	NR	NR	NR
[38]	Lee et al.	NR	NR	NR	NR	in the sclerotherapy group: 41.3%, in the LPS group: 36.1%, in the control group: 38.8%; *p* = NS	in the sclerotherapy group: 40.7%, in the LPS group: 33.3%, in the control group:33.3%; *p* = NS
[39]	Miquel et al.	NR	in the sclerotherapy group: 43.3%, in the control group: 23.2%; *p* = 0.01	NR	NR	in the sclerotherapy group: 37.3% in the control group: 15.9%; *p* = 0.01	in the sclerotherapy group: 31.3%, in the control group: 14.5%; *p* = 0.03
[43]	Yazbeck et al.	NR	NR	in the sclerotherapy group: 48.3%, in the LPS group: 19.2%; *p* = 0.04	in the sclerotherapy group: 55.2%, in the LPS group: 26.95; *p* = 0.03	NR	NR

Abbreviations: LPS—laparoscopic cystectomy; NR—not reported; NS—not significant.

**Table 5:** Newcastle–Ottawa scale assessment of the included studies.

Study	Selection	Comparability	Exposure	Total Score
Aflatoonian et al. 2013 [28]	NA	NA	NA	NA
Aflatoonian et al. 2020 [29]	3	1	2	6
Alborzi et al. 2021 [30]	2	2	2	6
André et al. 2011 [31]	3	2	2	7
Anvari et al. 2023 [32]	3	1	3	7
Begum et al. 2015 [33]	3	1	3	7
Hsieh et al. 2009 [34]	3	2	3	8
Huang et al. 2021 [35]	3	1	3	7
Ikuta et al. 2006 [36]	3	2	3	8
Koike et al. 2002 [37]	3	1	3	7
Lee et al. 2014 [38]	4	1	3	8
Miquel et al. 2020 [39]	3	1	2	6
Noma et al. 2001 [40]	3	1	3	7
Tehrani et al. 2022 [41]	NA	NA	NA	NA
Vaduva et al. 2023 [42]	3	1	3	7
Yazbeck et al. 2009 [43]	4	1	3	8

Abbreviations: NA—not assessed.

## Data Availability

Not applicable.
